# Nuragic Working Tools Characterization with Corrosion Layer Determinations

**DOI:** 10.3390/ma15113879

**Published:** 2022-05-29

**Authors:** Marta Porcaro, Anna Depalmas, Sergio Lins, Claudio Bulla, Matteo Pischedda, Antonio Brunetti

**Affiliations:** 1 Dipartimento di Chimica e Farmacia, Università degli Studi di Sassari, 07100 Sassari, Italy; mporcaro@uniss.it; 2 Dipartimento di Scienze Umanistiche e Sociali, Università degli Studi di Sassari, 07100 Sassari, Italy; cbulla@uniss.it (C.B.); matteopischedda07@gmail.com (M.P.); 3 Dipartimento di Scienze di Base e Applicate per L’ingegneria, Università degli Studi di Roma “La Sapienza”, 00161 Rome, Italy; sergio.lins@roma3.infn.it; 4 Dipartimento di Scienze Biomediche, Università degli Studi di Sassari, 07100 Sassari, Italy

**Keywords:** bronze corrosion, cultural heritage, XRF, Monte Carlo simulations

## Abstract

From the availability of metals to the technology and tools needed to transform them, roughly every civilization in the Mediterranean basin has a metalsmith story. Many of the objects produced by them share a few peculiar characteristics, usages, or even shapes. In this scenario, a class of objects that can be clustered by their usage, i.e., working tools, stands out from the crowd. For this study, a set of working tools from the Nuragic civilization (Sardinia, Italy) was researched with a non-destructive technique: X-ray Fluorescence (XRF). A quantification of the chemical species present in their alloy was obtained with the use of Monte Carlo simulations. The XRMC package, used for the simulations, managed for the first time to reproduce very complex corrosion layers and to thoroughly characterize them from a chemical perspective. The obtained results were discussed and compared to other results reported in the literature.

## 1. Introduction

Between the 17th and 7th centuries BC, a period that corresponds to both the so-called Nuragic Age (17th–13th centuries BC) and the Final Bronze/Early Iron Ages (12th–7th centuries BC), Sardinia was characterized by a great development of metallurgy with a wide production of bronze artifacts, the number and types of which only increased in time, reaching its apogee between the end of the Bronze Age and the Early Iron Age [1].

Its metallurgical products included work tools, weapons, and objects of high artistic value, such as vessels, ritual objects, small models of boats (navicelle), and small bronze statuettes–better known as bronzetti. A large number of artifacts found in Sardinia and other zones of the Mediterranean basin is clear evidence of both an autonomous production capacity and a strong interaction with other populations.

**Figure 1:**
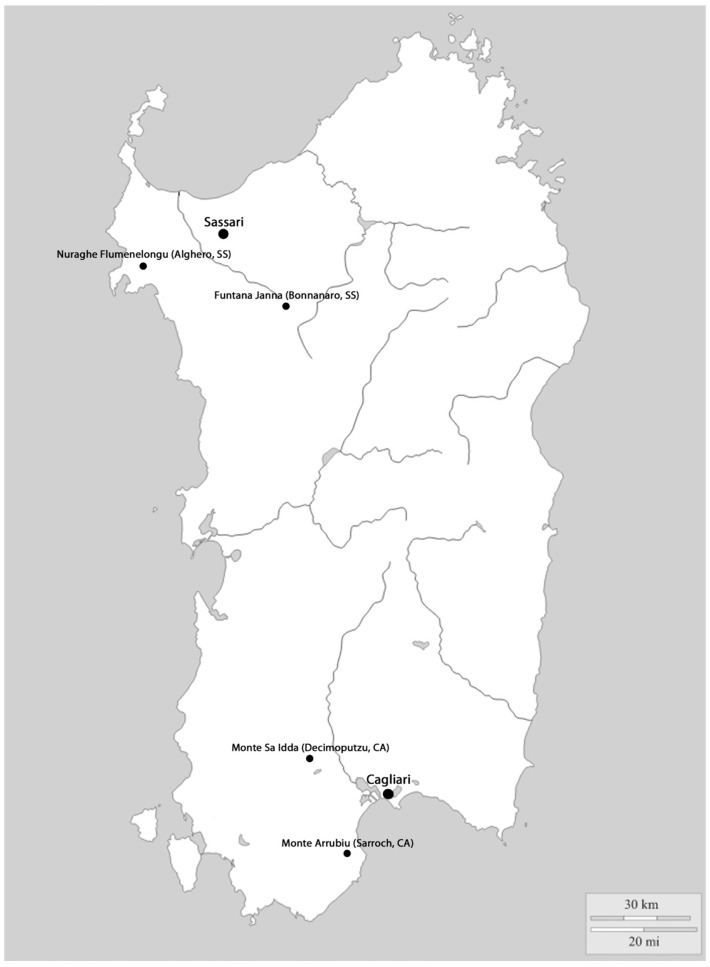
Map of Sardinia with indications of the sites where axes were found.

While Nuragic Sardinia’s most-known bronze artifacts, the navicelle and bronzetti, have been widely studied, other metalworks from the time of their production, such as working tools, have not received the same attention. Despite this, such “common” objects were produced in many different areas and by different populations in a way that a detailed comparison could yield important information about different metallurgic methods and the specific uses of such tools. For example, it could yield important information about the well-recognized link between many of the Sardinian artifacts found in several hoards and the Iberian Peninsula’s metallurgical production. For all these reasons, the present study focused on an analysis of a set of working tools from hoards discovered at Monte Sa Idda (Decimoputzu, CA) [2], Monte Arrubiu (Sarroch, CA) [3], Funtana Janna (Bonnanaro, SS) [4], and Nuraghe Flumenelongu (Alghero, SS; Figure 1) [5].

A bronze hoard from Monte Sa Idda was found inside a vessel comprising 92 artifacts, making Monte Sa Idda’s hoard one of the most important bronze collections of Nuragic Sardinia. In contrast, Monte Arrubiu’s hoard has only 12 bronze artifacts, including a few pieces in which influence from the Iberian Peninsula is evident, while Nuraghe Flumenelongu yielded a rich hoard consisting of seven artifacts and 32 plain ingots. Even in the latter case, a contextual presence of Iberian and peninsular forms can be observed. Finally, the Funtana Janna hoard includes Iberian and “Cypriot” models.

**Figure 2:**
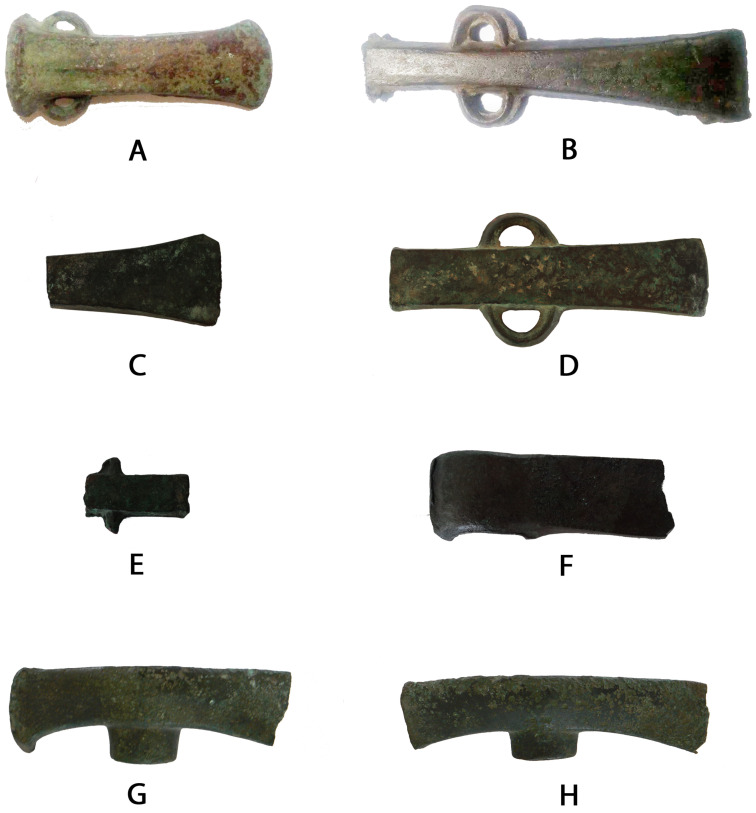
Images of the analyzed axes. Monte Sa Idda (Decimoputzu, CA): (**A**) cannon axe (#36265); Monte Arrubiu (Sarroch, CA): (**B**) flat axe with lateral loops (#39957); Nuraghe Flumenelongu (Alghero, SS): (**C**) fragment of a flat axe with lateral loops (#10273), (**D**) flat axe with lateral loops (#10271), (**E**) fragment of a flat axe with lateral spikes (#10275), and (**F**) shaft-hole axe (#10272); and Funtana Janna (Bonnanaro, SS): (**G**) double axe (#10712) and (**H**) double axe (#10713).

Despite the large number of artifacts described, not all of them are accessible. Only nine representative artifacts, listed in Table 1, have been analyzed: one flat axe with lateral loops from Monte Arrubiu (#39957); another flat axe with lateral loops (#10271) and a fragment of a flat axe (#10273)—both probably of the same type—from Nuraghe Flumenelongu; a fragment of a flat axe with lateral spikes (#10275) from Nuraghe Flumenelongu; two cannon axes (#36264 and #36265) from Monte Sa Idda; two double axes (#10712 and #10713)—reused as hammers—from Funtana Janna; and finally, a shaft-hole axe from Nuraghe Flumenelongu (Figure 2).

Some characteristic shapes of working tools are flat axes with lateral loops, axes with lateral spikes, and cannon axes with loops, which were produced on the Iberian peninsula. Double axes belong to a family of artifacts frequently compared to Cypriot production; the similarities are in the line with only more general features and not specific aspects. Shaft-hole axes, such as sample #10272, are extremely rare in Sardinia but rather common on the Italian peninsula, which confirms a trade relationship between Sardinia and continental Italy.

The above-listed artifacts were examined with the X-ray fluorescence technique (XRF). It is a well-known spectroscopic technique that, due to an intrinsic non-destructiveness, is commonly used for the investigation of cultural heritage objects [6]. Moreover, the instrumentation required is very portable, making it possible to examine objects that are not allowed to leave the tutelage of a museum, such as the samples described herein. A determination of the objects’ alloy composition was done with Monte Carlo simulations, which provide higher accuracies than other traditional methods. The entire protocol is discussed in the following section.

## 2. Materials and Methods

This work focused on the study of nine Sardinian axes preserved at the National Archaeological Museum of Cagliari and the National Archaeological Museum G.A. Sanna di Sassari. The samples came from different archaeological contexts and have different morphologies, as specified in Table 1.

As mentioned before, the samples were investigated using the XRF technique. It is based on the interaction of X-ray radiation with a sample. One of the consequences of this interaction is the production of X-ray photons called fluorescence photons emitted at fixed energies, each of them linked to a specific chemical element. Besides the fluorescence effect, another two factors must be considered for the range of energies normally used in XRF: the photoelectric effect (composing the peaks), and the incoherent effect (background). An elastic effect is usually negligible for the geometry used and does not change the energy of the photons.

**Figure 3:**
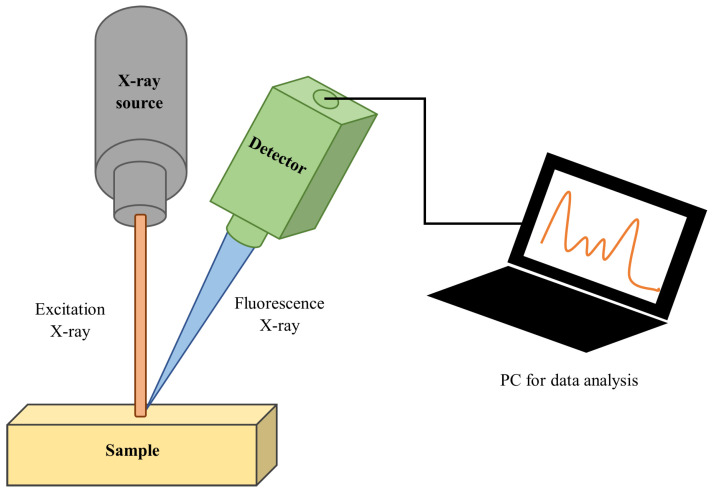
Schematic representation of a typical XRF experiment.

These effects, when combined, contribute to form the output of an XRF measurement (see Figure 3) called an X-ray spectrum. It is usually represented as a plot of a number of photons detected versus their energy. This spectrum can be divided into two main parts: a background (or continuum) and peaks. The former is essentially caused by scattered photons while the latter comes from the emission of X-ray fluorescence photons. Although the photons forming the background cannot be directly associated with a specific chemical element, they can be used to estimate which “chemical part” of a sample does not produce X-ray fluorescence. Thus, with a visual inspection of the X-ray spectrum, it is possible to estimate (roughly) the composition of a sample. This estimation can be refined by using several algorithmic procedures [7,8,9,10,11,12,13,14,15,16,17,18,19,20,21]. We used a Monte Carlo simulation of the real experiment for this scope.

A Monte Carlo simulation (MCS) is a probabilistic procedure that is used when a problem cannot be afforded in analytical ways. In the case of XRF measurements, MCS reproduces the real experiment by using a set of virtual photons that interact with a sample according to the real rules provided by the theory of nuclear interactions. In order to obtain a realistic simulation, two main steps are required: a faithful reproduction of the experimental setup and a guess model of the sample. The first step encompasses a geometric description of the experimental setup inside the Monte Carlo code and a semi-perfect reproduction of the spectrum emitted by the X-ray source, usually an X-ray tube. After this, a simulation of the experiment can be performed.

The simulated spectrum is compared to the measured one, and if differences are noticed, the structure and composition are changed and a new simulation is started. The cycle continues until the differences between the simulated and real spectra are negligible. They are initially checked via visual inspections and then refined by using a chi-squared test. There are many Monte Carlo software options able to simulate an XRF experiment, but many of them are too slow to be conveniently used in an XRF experiment simply because they are not specialized for XRF applications. Indeed, there is a small number of specialized codes that are fast enough to simulate an XRF experiment in a few minutes or less [17,21]. We used the software XRMC [19,20,21], which uses the *Xraylib* database [22,23]. Its use in a cultural heritage context has been discussed in depth elsewhere [24].

A combination of EDXRF and MC techniques provides essential information for the characterization of samples with the advantage of dismissing a preparation or the removal of the corrosion patina that normally covers the surface of a sample. XRMC allows us to ascertain the real composition of an alloy, taking into account overlying layers, whether they are formed by a corrosion/patina layer or a protective layer applied in a restoration. In fact, the layers covering a bulk (alloy) part are also estimated in terms of both composition and structure by using the MC simulation, making it possible to remove their contributions from the X-ray spectrum and leaving only the true alloy composition information.

All these factors combined allow one to reduce or even eliminate the surface enrichment effect that affects many alloys, bronze included. The only strong restraint in XRF measurements remains the patina thickness. If it is too thick (≈300 μm), and depending on its composition and density, a signal from the bulk cannot be detected. In this case, despite such limitations, XRF remains a precious tool for the chemical composition estimation of samples and often the only tool available.

When it comes to the MCS estimation error for elemental compositions, it is on average below 5 wt % for the main chemical elements that compose an alloy. It has nothing to do with the measurement itself but rather with uncertainties about the atomic parameters used for quantification, regardless of the quantification methods used. In any case, when we deal with ancient alloys, there is another source of error: a heterogeneity in an alloy’s composition inside a sample caused by the different mobilities of the alloy’s elements in any heat treatment. For this reason, it is mandatory to perform several measurements across an object at different positions. Error values are reported only if the differences between the measurement at different spots are larger than 5%; otherwise, the error must be considered around 5%.

The XRF instrument used for the measurements reported here was assembled at the University of Sassari and is composed of a mini-X X-ray tube with a silver anode working at 40 kV and an X-123 silicon drift detector (SDD) manufactured by Amptek^®^, Bedford, OH, USA. The geometry of the system is variable, with the detector commonly placed vertically from the surface of the object, 2–3 cm away, while the detector forms an angle of about 45° with the detector. The variable geometry allows one to take care of the shape and accessibility of the sample surface.

The MC code was run on an average laptop (with an AMD Ryzen 7 processor), and each simulation took about 20 s. As for the sample model used in the simulations, a couple of structures were tested: a two-layer structure (oxide alloy) and a three-layer structure (oxide–oxide alloy). The latter was used to simulate the corrosion model proposed by Robbiola et al. [25], which takes into account surface enrichment caused by corrosion. Tin enrichment is caused by the decuprification phenomenon as described in reference [25], which is a selective dissolution of copper and a low solubility and high stability of tin species. This was the first time that this kind of model was tested for a real bronze alloy with the package XRMC.

## 3. Results and Discussion

**Figure 4:**
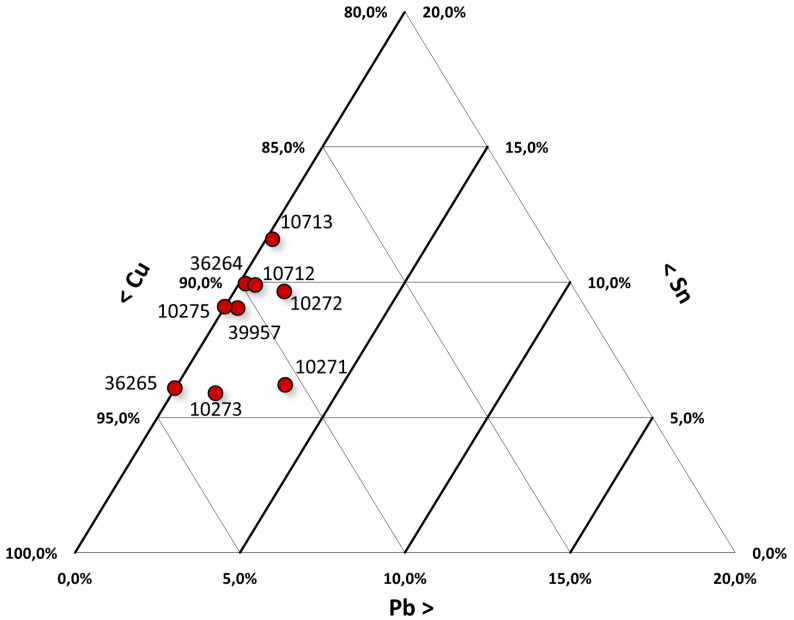
Ternary diagram for Cu-Sn-Pb of the composition of the axes.

The obtained results are summarized in Table 2, while the relationships among the various compositions are visible in the ternary diagram of Cu-Sn-Pb shown in Figure 4.

First, we will discuss the results in terms of sites and, when possible, compare them with other estimations previously published. Table 3 reports the oxidation layer structures for each sample. Samples #10712 and #10713 belong to the Funtana Janna hoard and were previously analyzed with the atomic absorption spectroscopy (AAS) [5] and neutron activation analysis (NAA) techniques [4]. A clear sign of material removal, as a result of the previous investigations, can be seen on the surfaces of the two samples. The surfaces in these regions were either partially re-oxidized (#10712) or strongly oxidized (#10713). XRF measurements were conducted at both the oxidized and pristine (uncleaned) zones on the samples’ surfaces. The composition obtained at the oxidized region from sample #10712 was obtained with a two-layer model. The MC simulation output was in good agreement with the values obtained with the AAS and NAA methods: copper (86% AAS, 82.75% NAA, and 85.36% XRF-MC), tin (11% AAS, 12.5% NAA, and 9.3% XRF-MC), and lead (0.91% AAS, 0.24% NAA, and 0.53% XRF-MC).

In the case of the unclean zones, the two-layer model failed, yielding a tin content value of about 19 wt % even if the simulated spectrum appeared to be a good reproduction of the measured one. Thus, according to reference [25], a three-layer model was tested for which the inner oxide layer was essentially composed of tin. This model yielded a better score and better agreement with the measured spectrum in comparison to the two-layered model. The concentration of tin decreased to 10.0 wt % in good agreement with the other destructive measurements reported. This result can be considered an achievement for the XRF technique since for the first time, the limitations of its quantification capabilities, caused by the migration of chemical elements to the surface or corrosion-product layer effects, were determined and overcome.

The above-described procedure was applied to the other samples as well, but in the case of the Flumenelongu samples, the two-layer model gave the best results. Moreover, for comparison purposes, only the NAA results are available for this set of samples. The XRF-MC method showed virtually the same values obtained with the NAA [4] technique except for an overestimation of the lead concentration in sample #10273 (0.93 wt % NAA against 1.5 wt % XRF). Previous XRF measurements, supposedly performed with standard quantification techniques [26] and where the sample was considered a single-layer structure, are available for the Flumenelongu samples analyzed herein. Even though the patina was probably removed for the previous XRF measurements mentioned, the tin and lead concentrations that were obtained were always higher with respect to those obtained with the XRF-MC method and NAA. This testifies to the superior quality of XRF-MC against traditional XRF quantifications.

In the case of the Monte Sa Idda hoard, there is no data from other quantitative techniques and no comparisons can be done. The samples from the Monte Sa Idda are made of binary bronze (Cu-Sn), all with similar compositions (Table 2): Cu 88–90 wt %, Sn 9–10 wt %, and Pb 0.2–0.4 wt %. These samples are also quite similar to the previous ones and can be considered binary Cu-Sn alloys. For all the samples from this hoard, the three-layer model outperformed the two-layer model.

**Figure 5:**
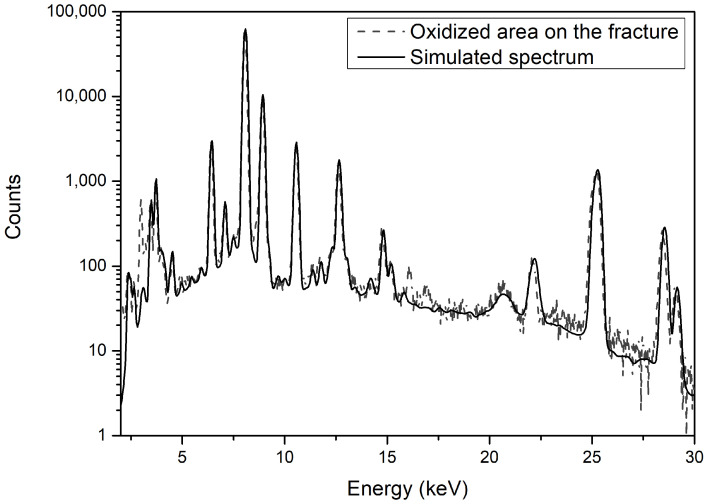
XRF spectrum of sample #10712’s oxidized area on the fracture (dashed line) and MC simulation (solid line).

These results represent, in our opinion, a big advance in XRF simulations, overcoming the problem of surface enrichment. However, it is mandatory to discuss the limits of this quantification. In fact, a three-layered structure is quite a complex model with a large number of variables, i.e., the thicknesses of the layers and their chemical compositions. This means that at first, more than one solution can be found. In order to test this hypothesis, several combinations of layer thicknesses and compositions were tested, starting around the values of the best fit. The idea was to find the maximum changes that did not introduce differences into the simulated spectrum; in other words, we looked for the method’s sensitivity. In the case of sample #10713, the thickness of the tin-rich layer decreased by 25% with respect to the best solution (from 10 μm thick down to 7 μm), and the concentrations in all the layers were changed in order to obtain the best fit (in this case, a copy of the best solution—one simulated spectrum of sample #10712—is shown in Figure 5). In the latter case, the tin concentration in the bulk increased from 11.8% to 12.5% (a less than 10% variation), copper net changes were lesser (1%), and the lead concentration increased from 0.2% to 0.25%. It is worth noting that NAA and AAS measurements gave a spread of tin concentration for this sample between 11.6% and 12.8%, so even with the 10% variation in tin content seen in the XRF-MC results, they were still compatible with those obtained with more precise but destructive techniques. For changes in thicknesses above 25%, the fit worsened.

Regarding the metallurgical aspect and considering all the samples, they are mainly formed from copper, tin, and, in some cases, lead (Table 2). The relationships among the various compositions are visible in the ternary diagram of Cu-Sn-Pb (Figure 4). Only one of the nine axes has a lead concentration exceeding 2 wt %: sample #10271, with a 3.2 wt % lead content. The presence of lead is not unusual in bronze alloys. It can be found both as an impurity, due to the use of certain minerals for smelting, and as a voluntary addition, since at contents around 2 wt %, it improves the fluidity of a casting, lowering the melting point. However, if the concentration increases, even up to only 3–4 wt %, the mechanical properties are lost, the hardness decreases, and the brittleness of the metal increases [27,28]. As for the remaining eight axes, it is difficult to assume that the addition of lead was intentional. The element was found in concentrations lower than 2 wt %. It is most likely that its presence came from the minerals used for the extraction of metals.

In addition to the main elements, iron, arsenic, and zinc were identified in some samples; they are also considered to likely be natural impurities from the minerals. The presence of iron inside the samples can provide information on the melting process and technological knowledge. Quantities around 0.05 wt % are typical of the first processes performed in conditions of poor reduction. However, percentages that exceed 0.3 wt % are an indication of more advanced metalworking skills [28].

**Figure 6:**
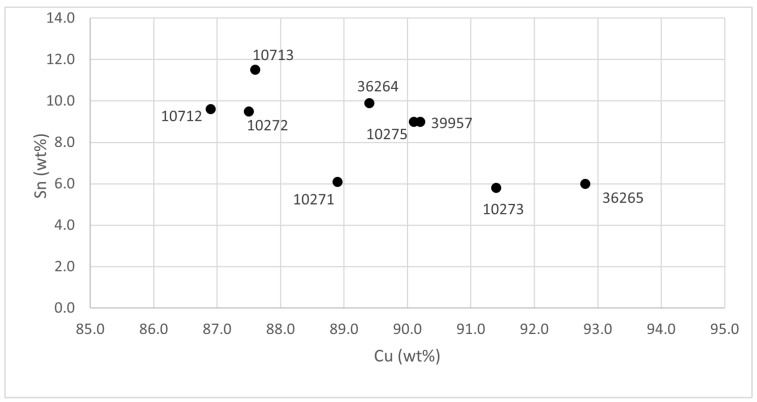
Cu/Sn diagram.

Bronzes can be classified according to their tin contents: (1) if the tin content is below 17 wt %, the alloy can be cold-worked and annealed; (2) if the tin content is between 17 and 19 wt %, the alloy is neither hot- nor cold-workable; (3) if the tin content exceeds 19 wt %, the bronze is workable only when hot and is almost always cast [27]. All the samples analyzed belong to the “category” of low-tin-content bronzes, with varying tin contents of between 5 and 11 wt % (Figure 6). This content is in line with the alloys found in Sardinia in the Bronze and Early Iron Ages [29].

**Figure 7:**
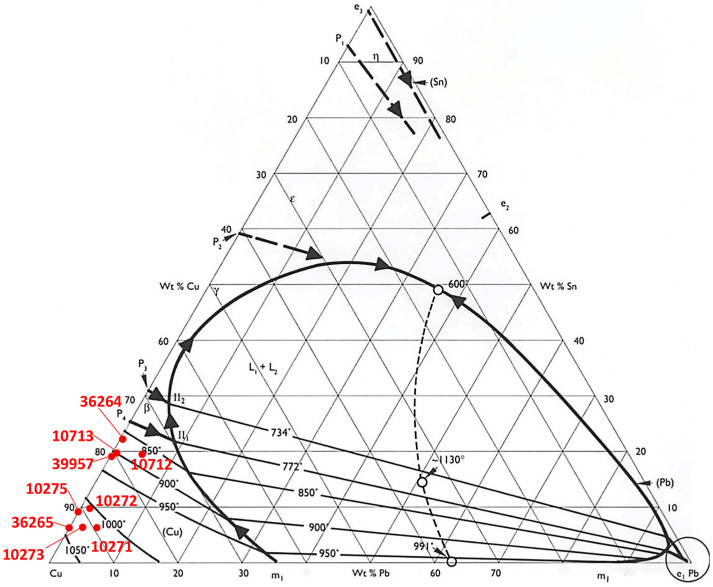
Cu–Sn–Pb ternary-phase diagram of the axes (red dots) indicating the melting temperatures reached.

Usually, tin was added to copper to strengthen the alloy, providing a greater tensile strength (see the bronze-phase diagram in Figure 7), but excessive quantities render the metal too difficult to work [1,28]; since axes are tools, the mechanical characteristics and a good resistance are fundamental.

For all the axes, it was possible to identify the presence of a surface-corrosion layer that varies between 20 and 85 μm. This layer can be studied to find fundamental information that may help understand the burial conditions and the corrosion processes to which an artifact has been exposed over time [25,30]. Elements, such as calcium, sulfur, and chlorine, were found in the corrosion patinas of some of the samples; their presence probably derived from the surface enrichment of elements present in the soil.

## 4. Conclusions

This research article concerns an XRF study of some Sardinian protohistoric tools. The main goal was to obtain some quantitative data about their compositions, which allows them to be compared to other similar objects produced in the Mediterranean basin and thus adds new data to the study of the region’s metallurgy. To reach this goal, a new approach to the use of XRF with Monte Carlo simulations was used. The novelty was not in the method itself since it has been used and published in several previous articles but in the digital model used for representing the sample. XRF measurements are superficial techniques that suffer from the tin and lead surface-enrichment effect typically present in bronze alloys. Until now, the effect has produced a strong overestimation of tin and lead concentrations in XRF quantitative measurements. Here, for the first time, this enrichment effect was modeled according to a structure described by Robbiola. The new model has estimated values in excellent agreement with other more precise and destructive techniques, such as NAA and AAS. In this sense, this research opens new perspectives on the application of XRF for faithful estimations of the compositions of bronze alloys.

## Figures and Tables

**Table 1:** List of analyzed samples with their inventory numbers, places of conservation, places of discovery, and artifact types.

Inventory Number	Museum	Location of Discovery	Typology
36264	Cagliari	Monte Sa Idda	Cannon axe
36265	Cagliari	Monte Sa Idda	Cannon axe
39957	Cagliari	Monte Arrubiu	Flat axe with lateral loops
10271	Sassari	Nuraghe Flumenelongu	Flat axe with lateral loops
10272	Sassari	Nuraghe Flumenelongu	Shaft-hole axe
10273	Sassari	Nuraghe Flumenelongu	Likely fragment of a flat axe with lateral loops
10275	Sassari	Nuraghe Flumenelongu	Fragment of a flat axe with lateral spikes
10712	Sassari	Funtana Janna	Double axe
10713	Sassari	Funtana Janna	Double axe

**Table 2:** Quantitative data expressed in weight percentages (wt %). Only the elements present in quantities greater than 0.5 wt % are reported.

Inventory Number	Copper (Cu %)	Tin (Sn %)	Lead (Pb %)	Zinc (Zn %)	Arsenic (As %)	Iron (Fe %)
36264	89.4	9.9	-	-	-	-
36265	92.8	6.0	-	-	-	0.7
39957	90.2	9.0	-	-	-	-
10271	88.9	6.1	3.2	0.6	0.5	0.5
10272	87.5	9.5	1.5	0.5	0.5	-
10273	91.4	5.8	1.3	-	-	-
10275	90.1	9.0	-	-	-	-
10712	86.9	9.6	0.5	-	-	-
10713	87.6	11.5	-	-	-	0.5

**Table 3:** Oxidation layer structure obtained.

Inventory Number	Bronze Patina Layer (µm)	Enrichment Layer (µm)
36264	25	10
36265	55	-
39957	30	10
10271	30	-
10272	20	-
10273	30	-
10275	25	-
10712	70	15
10713	25	10

## Data Availability

The data that were generated are available upon request from the authors.
